# Drug consumption rooms (DCRs) as a setting to address hepatitis C – findings from an international online survey

**DOI:** 10.1186/s41124-018-0035-6

**Published:** 2018-08-22

**Authors:** Vendula Belackova, Allison M. Salmon, Eberhard Schatz, Marianne Jauncey

**Affiliations:** 1 Uniting Sydney Medically Supervised Injecting Centre (MSIC), Sydney, Australia; 2 Correlation network, Foundation De Regenboog Groep, Amsterdam, The Netherlands; 3 School of Public Health and Community Medicine, University of New South Wales, Sydney, NSW Australia

**Keywords:** Drug consumption rooms, Supervised injecting facilities, Hepatitis C testing, Hepatitis C support services, Hepatitis C treatment, People who inject drugs

## Abstract

**Background:**

Prevalence of Hepatitis C Virus (HCV) among people who inject drugs (PWID) is high. Risky injecting behaviours have been found to decrease in drug consumption rooms (DCRs) and supervised injecting facilities (SIFs), yet HCV prevention and treatment in these settings have not been extensively explored.

**Methods:**

To determine the range and scope of HCV prevention and treatment options in these services, we assessed DCR/SIF operational features, their clients’ characteristics and the HCV-related services they provide. A comprehensive online survey was sent to the managers of the 91 DCRs/SIFs that were operating globally as of September 2016. A descriptive cross-country analysis of the main DCR/SIF characteristics was conducted and bivariate logistic models were used to assess factors associated with enhanced HCV service provision.

**Results:**

Forty-nine valid responses were retrieved from DCRs/SIFs in all countries where they were established at the time of the survey (Australia, Canada, Denmark, France, Germany, Luxembourg, Netherlands, Norway, Spain and Switzerland). Internationally, the operational capacities of DCRs/SIFs varied in terms of funding, location, size and staffing, but their clients all shared common features of vulnerability and marginalisation. Estimated HCV prevalence rates were around 60%. Among a range of health and social services and referrals to other programs, most DCRs/SIFs provided HCV testing onsite (65%) and/or offered liver monitoring or disease management (54%). HCV treatment onsite was offered or was planned to be offered by 21% of DCRs/SIFs. HCV testing onsite was associated with provision of other services addressing blood-borne diseases and HCV treatment was linked to the provision of OST. HCV disease management was associated with employing a nurse at a DCR/SIF and HCV treatment was associated with employing a medical doctor.

**Conclusions:**

DCRs/SIFs offer easy-to-access HCV-related services for PWID. The availability of onsite medical professionals and provision of support and education to non-medical staff are key to enhanced provision of HCV-related services in DCRs/SIFs. Funding and support for HCV treatment at the community level, via low-threshold services such as DCRs/SIFs, are worthy of action.

## Background

Drug consumption rooms (DCRs) / safe injecting facilities (SIFs) provide hygienic environments in which people who use/inject drugs (PWUD/PWID) can administer illegal drugs under the supervision of a health care professional, a trained allied service provider, or a peer (i.e., person who formerly used or currently uses illegal drugs), without the risk of arrest for drug possession [1]. DCRs/SIFs are evidence-based harm reduction interventions [2] with demonstrated effectiveness [3–11]. DCRs/SIFs generally target the most marginalised populations of people who use drugs (PWUD), people who inject drugs (PWID), and high-risk drug users (HRDU) [12, 13]. Studies among PWID have found that Hepatitis C Virus (HCV) prevalence at baseline is higher among DCR/SIF attendees than non-attendees [14], with daily attendees at greatest risk [15].

As such, DCRs/SIFs have the capacity to diagnose HCV among their clients in a timely way [16]. Several studies have documented the impact of DCRs/SIFs on HCV and HIV notifications [17–19], proving them cost-effective [20–22]. While decreases in risky injecting behaviour have been observed as an outcome of DCRs/SIFs attendance [23–26], HCV prevention and treatment in these settings has not been adequately explored in a cross-national context.

Recent research has demonstrated that high completion rates of HCV treatment among PWID [27], low rates of re-infection [28] and the provision of HCV treatment to active PWID are both effective and cost-effective [29, 30]. Adherence and response to the new, direct-acting antiviral agents (DAAs) therapy among PWID receiving opioid substitution treatment (OST) is comparable with people who never injected drugs [31], however numerous factors impede PWID participation in HCV treatment, including health system-related barriers, unstable housing or expectations of treatment side effects. Harm reduction services for PWID are an obvious avenue to circumventing some of these barriers [32] and can provide treatment-as-prevention [33], particularly to those clients attending on a daily basis or their “frequent attenders” [17, 34].

To date, surveys have not addressed HCV prevention and treatment among DCRs/SIFs clients [35, 36]. This study explored the range and scope of HCV-related services currently provided by DCRs/SIFs internationally, including HCV education, testing, disease management and treatment. We also explored their operational features and examined which factors were associated with the provision of enhanced HCV-related services at DCRs/SIFs.

## Methods

To assess the range and scope of HCV-related services currently provided by the 91 DCRs/SIFs operating in 10 countries across the world at the time of this study (September – December 2016), an online survey was conducted. An invitation to participate in the study was sent to the mailing list of the International Network of Drug Consumption Rooms and of the Correlation Network (European Network for Social Inclusion and Health). The initial email requested a manager (or person in a similar position) completed the survey on behalf of the organisation. Managers were also asked to decline participation on behalf of their organization if they decided not to participate. Requests to participate were emailed three times to those who did not fill in the survey or did not respond (in October, November and early December 2016).

Survey questions focused on the DCRs/SIFs organisational structure and environment, staff composition and general client characteristics (18 items). Data on client characteristics were requested as an estimate in aggregated form. HCV-specific questions focused on (i) HCV-related onsite interventions and referrals (18 items), (ii) DCRs/SIFs client characteristics in relation to HCV (2 items), and (iii) barriers and facilitators to the expansion of HCV-related services (2 items). Open-ended questions like “other” or “please, specify” where included. The survey was distributed in English. German translation was available and when completed, answers were translated back to English and entered into the online tool by the study team.

The survey took approximately 45 min to complete. To facilitate survey completion, all answers were made non-compulsory, resulting in a different number of responses in each question. All participants provided consent and could withdraw from the study at any time. Ethics approval was granted by the South Eastern Sydney Local Health District Human Research Ethics Committee (16/258 LNR/16/POWH/482).

### Study sample

The project used an exhaustive sampling method (i.e. approached all DCRs operating at the time of the survey). A total of 91 DCRs/SIFs from ten countries were invited to participate, namely from the Netherlands (*n* = 30 – number of DCRs in operation), Switzerland (*n* = 13), Germany (*n* = 24), Spain (*n* = 13), Norway (*n* = 1), Denmark (*n* = 5), Luxembourg (*n* = 1), Australia (*n* = 1), Canada (*n* = 1), and France (*n* = 2). In total, 86 survey responses were collected and from those, 37 were excluded from the analysis. Ten were excluded because they declined participation, nine due to duplicate responses, one response was from someone who wasn’t eligible to take part in the study (not a DCR representative) and 17 were excluded as there was missing data for greater than 50% of answers. The remaining valid responses (*n* = 49) represented 54% of the DCRs/SIFs in operation at the time of the study.

Among the survey responses that were included in the analysis, each country was represented, i.e. the Netherlands (*n* = 8), Switzerland (*n* = 7), Germany (*n* = 17), Spain (*n* = 9), Norway (*n* = 1), Denmark (*n* = 2), Luxembourg (*n* = 1), Australia (*n* = 1), Canada (*n* = 1) and France (*n* = 2) (Table 1). Responses were grouped according to DCF/SIF country of operation. The countries where only a few DCRs/SIFs operated (< 5) were included in the “remaining countries” category (*n* = 8).

**Table 1:** DCRs/ SIFs operating internationally and those who responded to the survey

	No of DCRs operating (September 2016)	No of DCRs participating in the study	Participation rate
Netherlands	20	8	40%
Switzerland	18	7	39%
Germany	26	17	65%
Spain	15	9	60%
Remaining Countries	12	7	64%
Australia	1	1	100%
Canada*	1	1	100%
France	2	2	100%
Denmark	6	2	33%
Norway	1	1	100%
Luxembourg	1	1	100%
Total	91	49	54%

* For more recent developments in Canada, please see the Health Canada website dedicated to Supervised Consumption (https://www.canada.ca/en/health-canada/services/substance-abuse/supervised-consumption-sites)

### Data and analysis

Client characteristics and service provision data were analysed using descriptive analysis. T-test and chi2 test were used to assess the difference in the mean values (for continuous variables) and in proportions (for categorical variables) between each country of operation and all other countries.

To explore the factors associated with HCV service provision, three outcome variables were considered: (1) provision of HCV testing onsite – saliva, finger prick, both, or other test, (2) provision of liver health monitoring (e.g. via fibro-scanning) and/or provision of support for HCV disease management, (3) provision of HCV treatment onsite (“new” treatments, interferon, or both) and/or planning to provide HCV treatment in the future. Bivariate logistic regression models were fitted in order to explore the statistical significance and size/direction of the relationship between outcome variables and a set of variables pertaining to DCRs/SIFs operational characteristics (unadjusted odds ratios were calculated). A range of operational characteristics were included in the bivariate logistic models as independent factors (Table 2).

**Table 2:** Per-country analysis of DCR/SIF characteristics (country vs. other; chi2 test, if not stated otherwise)

	Netherlands *n* = 8	Switzerland *n* = 7	Germany *n* = 17	Spain *n* = 9	Remaining countries *n* = 8	Total *n* = 49
	proportion (%)	proportion (%)	proportion (%)	proportion (%)	proportion (%)	proportion (%)
Operational						
Operated by the government	4/8(50.0)	3/6(40.0)	2/14(14.3*)	4/9(44.4)	5/8(62.5)	18/45(40.0)
Funding from local government	8/8(100.0*)	3/5(60.0)	11/15(73.3)	6/9(66.7)	4/8(50.0)	32/45(71.1)
Co-located with another program	5/8(62.5)	4/6(66.7)	10/15(66.7)	3/9(33.3)	4/8(50.0)	26/46(56.5)
Stand-alone facility with other services nearby	0/8(0.0*)	2/6(33.3)	3/15(20.0)	4/9(44.4)	5/8(62.5*)	14/46(30.4)
Staff-Related						
Employing a nurse	4/8(50.0*)	6/6(100.0)	11/15(73.3)	8/9(88.9)	8/8(100.0)	37/46(80.4)
Employing a medical doctor	3/8(37.5)	0/6(0.0*)	7/15(46.7)	4/9(44.4)	6/8(75.0*)	20/46(43.5)
Employing peer workers	3/8(37.5)	2/6(33.3)	1/15(6.7)	1/9(11.1)	3/8(37.5)	10/46(21.7)
Number of paid staff on average day - n (mean country / other - t-test)	6(3/8*)	6(6/8)	13(8/7)	9(7/7)	8(11/7*)	42(7)
Client-Related						
Number of attendees per day - n (mean country / other - t-test)	7(16/130*)	5(158/100)	12(106/108)	8(109/107)	4(206/95*)	36(108)
% of clients tested for HCV - n (mean country / other - t-test)	5(59/72)	5(78/69)	14(77/67)	9(77/69)	8(54/75)	41(71)
% of clients estimated as HCV positive - n (mean country / other - t-test)	5(33/61**)	5(51/59)	15(57/58)	8(69/55*)	8(67/55)	41(58)
Service-Related						
Naloxone onsite	1/8(12.5)	1/6(16.7)	4/15(26.7)	8/9(88.9***)	3/8(37.5)	17/46(37.0)
HIV counselling onsite	1/8(12.5***)	3/6(50.0)	14/15(93.3*)	8/9(88.9)	6/8(75.0)	32/46(69.6)
HIV testing onsite	0/8(0.0***)	2/6(33.3)	9/15(60.0)	8/9(88.9*)	6/8(75.0)	25/46(54.4)
OST onsite	2/8(25.0)	0/6(0.0)	5/15(33.3)	3/9(33.3)	1/8(12.5)	11/46(23.9)
HBV vaccination onsite	1/8(12.5)	1/6(16.7)	7/15(46.7)	6/9(66.7)	4/8(50.0)	19/46(41.3)
Number of spaces for drug use - n (mean country / other - t-test)	7(10/12)	5(26/10***)	15(11/12)	8(5/13*)	6(13/12)	41(12)
HCV-Related services						
HCV testing onsite	2/7(28.6*)	4/7(57.1)	12/17(70.6)	8/9(88.9)	6/8(75.0)	32/48(66.7)
Liver monitoring or disease management onsite	2/7(28.6)	2/6(33.3)	11/17(64.7)	5/8(62.5)	5/8(62.5)	25/46(54.4)
HCV treatment onsite or plan to provide in the future	1/7(14.3)	0/6(0.0)	5/17(29.4)	1/9(11.1)	3/8(37.5)	10/47(21.3)

*statistically significant difference (country group vs. all other countries) on *p* < 0.05 level; **statistically significant difference on *p* < 0.01 level; ***statistically significant difference on *p* = 0.00 level

## Results

### Descriptive Characteristics

#### DCRs/SIFs operational features

Most DCRs/SIFs were operated by a not-for-profit organisation (30/45, 67%). Other DCRs/SIFs providers included local, regional or national governments (18/45, 40%), private entities (3/45, 7%), or via contracts with a government (2/45, 4%) or religious organisation (*n* = 1). Services were funded from a variety of sources, often more than one. Funding was primarily provided by local (32/45, 71%), state/regional (16/45, 36%) or national (6/45, 13%) governments. Other funding sources included charities, drug service subsidies, donations or confiscated proceeds of crime.

The DCRs/SIFs were most commonly located in the centre of town (34/46, 74%), near a major travel hub (25/46, 54%) or within the boundaries of an established street-based drug scene (22/46, 48%). One fifth operated a mobile service (among those, two DCRs/SIFs operated a mobile service only). More than half were co-located with other services (26/46, 57%) and in four cases, these were shelters or overnight housing programs. About one third were stand-alone facilities, with other services used by their clients located nearby (14/46, 30%).

#### DCR/SIF capacities and services

**Fig. 1:**
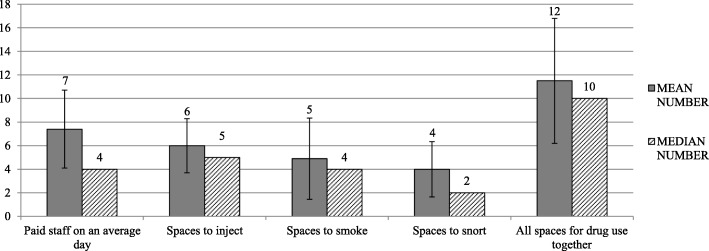
Operational capacities of DCRs/SIFs

The clinical staff at DCRs/SIFs included nurses (37/46, 80%), social workers (36/46, 78%), health educators/rescue workers (13/46, 35%) and medical doctors (20/46, 46%). Other professions employed by some DCRs/SIFs were psychiatrists, psychologists, case managers or researchers. Peers worked at one fifth and students / trainees in one sixth of the DCRs. About one third of DCRs/SIFs employed security personnel (15/46, 33%). Where estimated by the managers (*n* = 42), the average number of paid staff onsite on an average day was 7 (SD = 6.6, see Fig. 1 for details). A median of 10 spaces were offered for supervised drug consumption in DCRs/SIFs overall and the largest program had 63 spaces. See details of the operational capacities in Fig. 1.

**Fig. 2:**
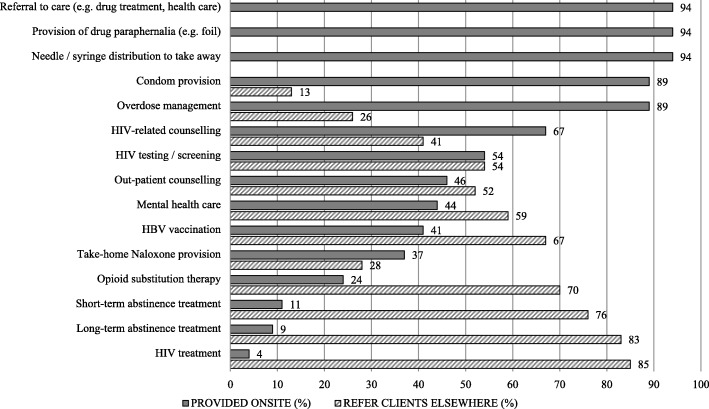
Health services provided onsite and referred to offsite services by the DCRs/SIFs (*n* = 46)

**Fig. 3:**
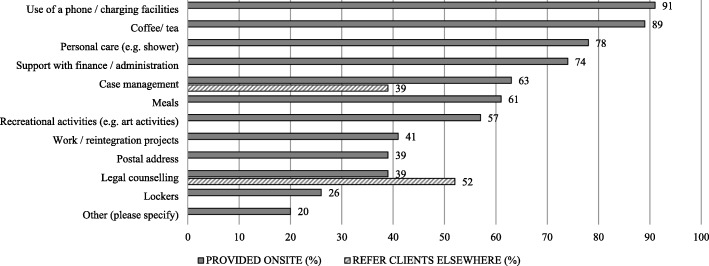
Social services provided onsite and referred to offsite services by the DCRs/SIFs (*n* = 46)

Beyond the supervision of the drug administration, a range of services composed the “core business” of DCRs/SIFs and these were distribution of needles and syringes for onsite and offsite use, onsite overdose management and provision of referrals to health and social services. The majority of DCRs/SIFs also provided onsite HIV-related counselling (31/46, 67%) and HIV testing (25/46, 54%). A SIFs provided/SIFs provided medical treatments onsite: OST (11/46, 24%), abstinence-oriented treatment (5/46, 11%), or HIV treatment (2/46, 4%). The health and the social services provided at DCRs/SIFs are summarized in Figures 2 and 3 respectively. It was common to refer clients elsewhere when services were not provided onsite.

#### HCV rates and other characteristics among DCR clients

A median of 78 visits were made at a DCR each day (mea*n* = 108, SD = 120, *n* = 36) and the largest number of visits made at a DCR on an average day was in Denmark (550).

Forty-one DCRs in the study provided estimates of the proportion of their clients who had been tested for HCV (median = 80%) and who were HCV positive (median = 60%). The highest rates of HCV positive clients (80% and more) were estimated in nine DCRs from across all European countries except for the Netherlands and France. The proportion of clients with HIV was 6% (estimated median).

**Fig. 4:**
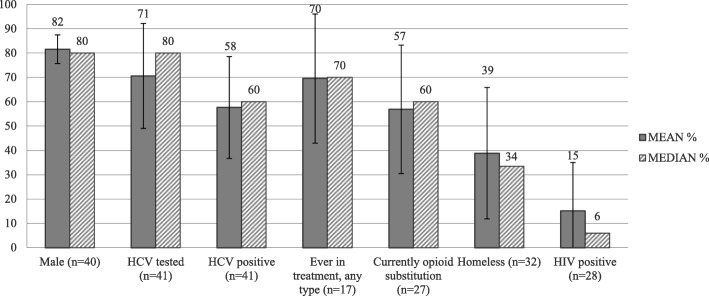
DCR/SIF client characteristics, estimates reported by survey participants

The median age of DCR/SIF clients was approximately 40 years old and most were male (median = 80%, *n* = 45). Most DCR/SIF clients were estimated to have ever entered drug treatment (median = 70%, *n* = 17) and to be on OST at the time of the survey (median = 60%, *n* = 27). About one third of clients (median 34%, *n* = 32) were estimated to be homeless. All characteristics of DCRs/SIFs clients who participated in the survey are summarized in Fig. 4.

#### HCV-specific services provided at DCRs/SIFs

In response to the HCV rates reported above and as a prevention measure, almost all DCRs/SIFs in the study indicated that they provided HCV-related education (46/49, 94%). This took various forms (educational materials, consultations) and focused on providing information about transmission routes, infection symptoms, testing or treatment options (Table 3). The majority of the DCRs/SIFs also offered HCV testing onsite (32/49, 65%). The testing was most commonly done through a blood sample, but also saliva and/or finger prick tests were performed. From those facilities that were not providing HCV testing onsite (*n* = 16), eight were planning to do so in the future and nine currently referred clients elsewhere.

**Table 3:** HCV education and testing provided onsite at DCRs/SIFs

Provided onsite		Form provided	
HCV education (multiple response)	*n* = 49	If HCV education provided onsite (multiple response)	*n* = 46
HCV prevention & transmission routes	94%	Brochures/pamphlets	90%
HCV testing	78%	Individual client consultations	88%
HCV infection symptoms	76%	Posters	69%
HCV treatment options	76%	Other (please specify)	20%
We don’t provide any HCV information	6%	Digital resources (e.g., videos, quizzes)	20%
		Group education sessions	18%
HCV testing onsite	*n* = 49	If HCV testing provided onsite (multiple response)	*n* = 32
Yes	65%	BLOOD SAMPLE taken from a vein	68%
No	33%	SALIVA / oral fluid	39%
Don’t know	2%	FINGER PRICK test	32%
		Other	10%

Almost all DCRs/SIFs referred their clients to HCV treatment programmes (Table 4). More than half (25/46, 54%) offered enhanced HCV services in the form of liver monitoring or disease management (Table 4). About half (24/47, 51%) of the DCRs/SIFs in the study planned to expand HCV referrals or the enhanced support services in the future. Only four DCRs/SIFs provided HCV treatment onsite at the time of the survey (8%), two of them in the form of DAAs. Six additional services were planning to provide HCV treatment in the near future: either new treatments (*n* = 1), interferon treatment (*n* = 1) or an unspecified type of treatment (*n* = 4).

**Table 4:** HCV support services and treatment provided onsite at DCRs/SIFs and availability of HCV treatment to DCR clients

	Provide onsite	Plan to expand in future
HCV support (multiple responses possible)	*n* = 46	*n* = 47
Yes, referral to other services that can provide HCV treatment	96%	36%
Yes, disease self-management support (e.g. healthy diet, obesity)	50%	15%
Yes, liver health/cirrhosis monitoring (e.g. fibro-scan, blood test)	24%	11%
No	15%	49%
HCV treatment (one response only)	*n* = 48	*n* = 39
Yes, new HCV treatment forms (DAAs)	4%	3%
Yes, interferon-based treatment	0%	3%
Yes, both treatment options	4%	0%
Other (provided details)	–	10%
Don’t know	8%	–
No	86%	84%

### Differences between countries

The Dutch DCRs/SIFs (*n* = 8 of the 49) differed from other countries in a number of ways: they were receiving funding from local government (chi2 = 3.95, *p* = 0.047), they did not offer stand-alone programs, and, they operated with fewer paid employees (chi2 = 6.36, *p* = 0.012) and fewer nurses (chi2 = 5.70, *p* = 0.017) than other services surveyed. They also reported a lower number of visitors per day (*t* = 2.38, *p* = 0.01), and provided a lower estimate of HCV-positive clients (*t* = 3.03, *p* = 0.002). The Dutch DCRs/SIFs were also less likely to provide onsite HIV services (chi2 = 11.53, *p* = 0.00) or HCV testing (chi2 = 5.35, *p* = 0.02) than those located elsewhere.

German DCRs/SIFs (*n* = 17) differed from those in other countries in that a lower proportion of them offered HIV counselling (chi2 = 5.94, *p* = 0.015). Also unique to German DCRs was that they were less likely to be operated by the government (chi2 = 5.60, *p* = 0.018).

The Spanish DCRs/SIFs offered onsite HIV testing more often than others (chi2 = 5.38, *p* = 0.02), and also over-performed with respect to providing Naloxone training onsite (chi2 = 12.95, *p* = 0.00). The Spanish DCRs/SIFs (*n* = 9) tended to have fewer drug consumption spaces (*t* = 1.97, *p* = 0.03) than other countries. Also, the Spanish DCRs/SIFs estimated a significantly higher proportion of HCV positive clients (*t* = -1.80, *p* = 0.04).

Contrastingly, a higher number of drug consumption spaces (*t* = -3.46, *p* = 0.000) was reported by the Swiss DCRs/SIFs (*n* = 7). At the same time, the Swiss programs were less likely to employ a medical doctor (chi2 = 5.31, *p* = 0.021).

In the group of “remaining countries” (*n* = 8), employing a medical doctor (chi2 = 3.95, *p* = 0.048) was more common than elsewhere, they employed a higher number of staff (*t* = -1.83, *p* = 0.038) and were more commonly operated as stand-alone facilities (chi2 = 4.70, *p* = 0.030). They also had a significantly higher number of attendees on an average day (*t* = -1.79, *p* = 0.041). See Table 2 for a more detailed cross-national comparison.

### Factors associated with provision of HCV services at DCRs/SIFs

HCV testing was conducted more frequently in DCRs that provided HIV testing (OR = 44.6, *p* = 0.00) or HBV vaccination (OR = 7.3, *p* = 0.01). Factors positively associated with HCV support (defined as provision of liver monitoring or disease management) onsite at the DCR/SIF were: onsite HIV counselling (OR = 4.1, *p* = 0.04), testing (OR = 7.0, *p* = 0.00) and HBV vaccination (OR = 6.7, *p* = 0.00), onsite OST (OR = 5.4, *p* = 0.03), and employment of a nurse (OR = 12.4, *p* = 0.01) or a medical doctor (OR = 8.0, *p* = 0.00). Current or future provision of HCV treatment onsite was significantly associated with employing a medical doctor (OR = 14.6, *p* = 0.00)  and marginally asssociated (OR = 4.3, *p* = 0.08) with provision of OST onsite (Table 5).

**Table 5:** Factors associated with HCV service provision at DCRs/SIFs - bi-variate logistic regression

	(1) Provide HCV testing onsite (*n* = 32; 665%) *OR (standard error), p-value*	(2) Provide liver monitoring or disease management onsite (*n* = 25; 51%) *OR (standard error), p-value*	(3) Provide HCV treatment onsite or planning to provide in near future (*n* = 10; 20%) *OR (standard error), p-value*
Operational			
Country (Netherlands)	0.15* (0.13), 0.02	1.48 (0.37), 0.11	1.39 (0.42), 0.26
Operated by the government	0.89 (0.58), 0.86	0.53 (0.33), 0.31	1.57 (1.23), 0.57
Funding from local government	0.55 (0.41), 0.41	0.76 (0.52), 0.69	0.64 (0.53), 0.59
Co-located with another program	1.38 (0.90), 0.61	1.67 (1.02), 0.40	2.84 (2.50), 0.21
Stand-alone facility with other services nearby	2.01 (1.51), 0.53	1.17 (0.76), 0.81	0.69 (0.62), 0.68
Staff-Related			
Employing a nurse	2.08 (1.60), 0.34	12.38** (13.92), 0.01	0.70 (0.64), 0.70
Employing a medical doctor	1.48 (0.98), 0.55	8.00*** (5.66), 0.00	14.58*** (16.41), 0.00
Employing peer workers	0.88 (0.70), 0.87	0.47 (0.34), 0.29	1.43 (1.31), 0.70
Higher than median number of paid staff on average day (> 4)	1.20 (0.80), 0.80	1.22 (0.77), 0.75	2.00 (1.62), 0.39
Client-Related			
Higher than median number of attendees per day (> 78)	1.42 (1.04), 0.63	0.62 (0.43), 0.49	2.14 (2.02), 0.41
Higher than median % of clients tested for HCV (> 80%)	1.98 (1.38), 0.32	0.38 (0.26), 0.15	0.51 (0.38), 0.36
Higher than median % of clients estimated as HCV positive (> 60%)	0.83 (0.57), 0.79	0.60 (0.40), 0.44	1.00 (0.75), 1.00
Service-Related			
Provide naloxone onsite	3.02 (2.25), 0.12	1.67 (1.07), 0.42	0.49 (0.43), 0.40
Provide HIV counselling onsite	2.57 (1.77), 0.17	4.09* (2.90), 0.04	3.36 (3.78), 0.23
Provide HIV testing onsite	44.57*** (50.06), 0.00	7.00*** (4.75), 0.00	2.84 (2.50), 0.21
Provide OST onsite	2.45 (2.11), 0.27	5.40* (4.62), 0.03	4.29 (3.52), 0.08
Provide HBV vaccination onsite	7.29** (6.15), 0.01	6.67*** (4.67), 0.00	1.47 (1.15), 0.62
Higher than median no of spaces for drug use (> 10)	0.63 (0.43), 0.49	0.62 (0.39), 0.44	2.97 (2.68), 0.21

*statistically significant difference (country group vs. all other countries) on *p* < 0.05 level; **statistically significant difference on *p* < 0.01 level; ***statistically significant difference on *p* = 0.00 level

When the managers were asked how they would spend any additional HCV-related funds, most said they would employ additional medical staff (24/46, 52%), develop client education around HCV (24/46, 52%) and/or provide additional staff training (21/46, 46%). Other responses included developing policies and procedures for staff, preparing educational material for clients or employing peer support workers. Conducting a needs assessment or developing referral pathways to a specialist were mentioned as well. Two organisations said that they would purchase a fibro-scan and one organisation mentioned that they would invest in advocacy to garner support to provide HCV treatment to “clandestine” persons.

When it comes to the reasons for not providing enhanced HCV-related services, some respondents indicated that HCV services were not part of the formal purpose of their facility (3/47, 6%). Other DCRs/SIFs mentioned that the site was already very busy or that the setting simply did not allow HCV services (*n* = 2), or that the DCRs/SIFs approach was non-medical and that current referral pathways worked well (*n* = 1). Other barriers included the high cost of rapid test kits (*n* = 1) or the fact that clients of the service were undocumented migrants who could not access treatment even if HCV positive.

## Discussion

This study explored the range and scope of HCV-related services currently provided by DCRs/SIFs internationally and examined associated factors. We found that prevention and management of blood-borne infections is a priority for the vast majority of the DCRs/SIFs that responded to this online survey. Most of them reported that they provided HCV-related education in some form and referred their clients to HCV testing and treatment. Over two thirds provided onsite HCV testing. Over half of those surveyed offered enhanced HCV support services that included liver-health monitoring or disease management. One fifth of the DCRs/SIFs in the study offered or planned to offer HCV treatment in the near future.

Current provision of enhanced HCV-related services was (with one exception) independent of the DCR/SIF country of origin, the DCR/SIF source of funding, any co-location with other programs, the size of the DCRs/SIFs or the actual HCV prevalence rates among their clients. Factors positively associated with HCV service provision were the provision of services related to blood-borne disease prevention and care, the provision of OST and the staff health and medical qualifications.

Internationally, the operational capacities of DCRs/SIFs vary, but their clients all share common features of marginalisation, including estimated HCV prevalence rates of about 60%. DCRs/SIFs are well positioned to support PWUD to access HCV-related services, given the ease of access and the established relations between staff and clients which support uptake and adherence to HCV treatment [37]. The fact that HCV testing was significantly more likely to occur in DCRs/SIFs that offered HIV testing, or that provided vaccination for HBV onsite, is of high importance for PWUD who often live with hepatitis and HIV co-infections [38].

In HCV management, non-invasive liver disease assessment (e.g. fibro-scanning) is recommended as a feasible method in out-patient settings for PWUDs, alongside individualised disease management within a multidisciplinary team [39]. Treatment as prevention can significantly reduce HCV prevalence among PWUD and while DAAs make this approach more feasible [39], combining it with OST improves its effectiveness and can reduce population-level HCV rates [40].

In the Netherlands, HCV incidence and chronicity rates among PWUD have been the lowest in the European region [41] and have been decreasing for some time [42] in response to intensive prevention and treatment efforts. The decreasing rates of drug injection in the Netherlands have likely contributed to this trend [43, 44]. It was unsurprising then that the Dutch DCRs/SIFs were less likely to provide HCV testing and related services pertaining to blood-borne diseases.

There were other per-country differences in DCR operational characteristics. For instance, it has long been part of the Dutch DCR concept to run smaller facilities in multiple neighbourhoods and to co-locate them with other services, rather than one large stand-alone facility in a central area [45]. In contrast, our findings show countries with few DCRs/SIFs are more likely to operate as stand-alone services (for example, the Medically Supervised Injecting Centre in Sydney, Australia).

Between 2017 and 2018, about thirty new DCRs/SIFs were approved in Canada (in addition to Insite in Vancouver) [46]. The operational, financial and public health considerations of establishing additional DCRs/SIFs seem to favor smaller facilities which are well-integrated within existing agencies [47]. This study indicated that stand-alone facilities require larger investments in personnel, including medical staff. The advantage of the co-located services is that they can support PWUD/PWID directly with relevant care onsite.

When looking at the determinants of HCV service provision, these don’t imply any causality and several other, unmeasured factors might play an important role. For instance, Australia has adopted universal access to HCV treatment, which is unprecedented internationally. Also, the Australian SIF employs a medical doctor. However, HCV treatment is not provided at the Sydney SIF, given the regulations around prescribing authority and it’s close cooperation with a nearby health service. On the other hand, the local-level funding of DCRs in the Netherlands may have led to substantial local-level variability in operating procedures and funding priorities, including HCV provision. Local policies play an obvious role in the level of HCV service provision but this study wasn’t designed to explore that in detail.

Low-threshold, harm reduction services such as DCRs/SIFs aim to “meet individuals where they are” in order to reduce individual and community-level harmful effects of drug use. Some clients might be systematically “falling through the net” of support services, leaving DCRs/SIFs the only programs they attend on a regular basis. This study found that HCV management and treatment were marginally more likely to be offered by DCRs/SIFs which also ran OST onsite. Unsurprisingly, the capacity to provide enhanced HCV care was closely related to the qualifications of DCRs/SIFs staff. Provision of liver monitoring or disease management were significantly associated with employing a nurse whereas HCV treatment was associated with employing a medical doctor. Enhancing staff qualifications via continuous education and greater engagement of health professionals in DCRs/SIFs operations will lead to more advanced HCV service provision and to continued engagement of the most vulnerable clients.

However, it needs to be acknowledged that hiring medical staff can represent a notable organisational and financial challenge for many DCRs/SIFs who are mostly funded outside of the general health funding schemes (e.g. local government budgets). Innovative protocols for the delivery of DAA treatments can include empowering existing staff via education and training to facilitate their cooperation with external medical professionals. In a recent Australian study, case managers who were supported by onsite nurses and medical doctors delivered DAA treatment in the community and achieved high completion rates [48]. This model can serve as an example for delivering flexible, patient-centred and cost-effective HCV treatment on community level. An additional barrier to implementing HCV treatments in DCRs might be the affordability of the medication. While the cost of medication is fully covered in Australia [49, 50], this has not been the situation in many low- and middle- income countries [51].

This study is limited by the relatively high non-response rate to the online survey (46%). Completing an online survey competes with other priorities and in some cases, filling in the survey was interrupted multiple times. In other instances, questions that required additional research into the DCR/SIF’s internal resources were skipped.

The DCRs/SIFs that did not participate in the survey (*n* = 52) may offer different services to specific client groups, depending on their location and purpose, or have lower operational capacity. Some DCRs/SIFs in the study mentioned that enhanced provision of HCV-related services was not a key purpose of their operation. It is possible that DCRs/SIFs with greater affinity for HCV service provision chose to answer the survey. Beyond this survey, there is no official source of information that could be used to cross-validate the representativity of the sample. An important limitation to this study is that the survey wasn’t available in all national languages (e.g. Spanish, Dutch, Danish or Norwegian) and misrepresentation of questions may have occurred.

## Conclusions

The high infection rates of HCV among the DCRs/SIFs clients and the fact that HCV treatment as prevention can significantly reduce HCV rates among the population of PWUD warrant the attention for DCRs/SIFs to be seen as a potential space for the provision of HCV related services worldwide.

With their ease of access (‘low threshold’) and the regularity with which some clients attend them, DCRs/SIFs offer an important entry point to HCV treatment as well as a candidate program for the provision of new, DAA treatments. DCRs/SIFs can offer easy-to-access services and a trusted environment that is crucial in achieving HCV treatment adherence.

The main steps forward to enhance the onsite provision of HCV-related support in DCRs/SIFs are increasing their capacity to accommodate onsite medical professionals and educating existing staff. Funding and support for HCV treatment at the community level, via low-threshold services such as DCRs/SIFs, is worthy of action.

## Abbreviations

DAA: Direct acting antivirals
DCR: Drug consumption room
HBV: Hepatitis B virus
HCV: Hepatitis C virus
HIV: Human immunodeficiency virus
OST: Opioid substitution treatment
PWID: People who inject drugs
PWUD: People who use drugs
SIF: Supervised injecting facility
